# Supplementary material for: Structural and functional dissection of the interplay between lipid and Notch binding by human Notch ligands
Source: EMBO J. 2017 Jun 1;36(15):2204–15. doi: 10.15252/embj.201796632 (PMC5538765; doi:10.15252/embj.201796632)
Supplement: Supplementary file 1 — Appendix [file EMBJ-36-2204-s001.pdf]

Appendix

1. Supplemental Methods
2. Extended structure determination procedures
3. Additional References

1. Supplemental Methods

Cloning

Codon optimized open reading frames (synthesized by GeneArt®, Life Technologies Ltd., Paisley, UK), with either their native signal peptide, or recommended BiP signal peptide (for secretion), were subcloned into expression vector pEXS2-2 (Expres2ion® Biotechnologies, Horsholm, Denmark) using EcoR I and Not I restriction sites. Inserts contained an upstream Kozak sequence and C-terminal 8 x Histidine tag. Constructs encoding Notch EGF11-13 also contained a PreScission protease site and a BirA tag between Notch and the C-terminal His-tag. All constructs used encoded Notch ligands and receptors from humans, and are detailed in table S1. All constructs were sequenced by Source BioScience (Nottingham, UK) prior to use. Jagged1 Del1Del2Asp140Ala contains a C54S mutation, deletion of residues 58-68 replaced with one serine, a D140A mutation, and deletion of residues 143-148 replaced with one glycine.

Protein	Domains	Residues
Jagged1	N-EGF3	32-337
Jagged2	N-EGF3	27-348
	N-EGF2	22-329
Delta-like1	N-EGF3	22-329
Delta-like3	N-EGF1	27-275
Delta-like4	N-EGF3	27-325
Notch1	EGF11-13	411-528
Notch2	EGF11-13	414-532

Appendix Table S1 – Constructs used in these studies.

Protein expression and production:

Constructs were stably transfected into S2 insect cells (Expres2ion® Biotechnologies) for recombinant protein expression. Selection was undertaken using Geneticin® (Life Technologies Ltd.) for a period of 26 days during which cell media (EX-CELL® 420, Sigma-Aldrich, Dorset, UK) was replaced and volume was expanded to 15 ml. After selection, cells were further expanded to 1L volume in CELLMASTER™ 850 cm^2 roller bottles to a cell density of up to 65 million / ml. Media containing recombinantly expressed protein was then filtered and loaded onto a cOmplete His-tag Purification Column (Roche Diagnostics, UK), for purification via His-tag. Following washing with 30 column volumes of 50 mM Tris pH 9.0, 5 mM Imidazole pH 8.0, 200 mM NaCl, 1 mM $CaCl_2$, proteins were eluted with 8 column volumes of buffer containing 250 mM Imidazole pH 8.0. Eluted proteins were dialysed overnight in 10 mM Tris pH 7.5, 200 mM NaCl, 1 mM $CaCl_2$ at 4°C. Proteins were concentrated and loaded onto a HiLoad S200 (for ligands) or S75 (for receptors) 26/600 prep grade size exclusion column (GE Healthcare, Waukesha, WI, USA) pre-equilibrated in 20 mM Tris pH 7.5, 200 mM NaCl, 1 mM $CaCl_2$. However both Jagged2 N-EGF2 and cleaved Notch2 EGF11-13 were gel filtered in 10 mM Tris pH 7.5, 150 mM NaCl, 2 mM $CaCl_2$ for crystallization. Both the C-terminal BirA and His tags in the Notch2 EGF11-13 construct were cleaved off for crystallization; the affinity purified protein was incubated with 0.5 mg of PreScission protease before dialysis overnight at 4°C. Non-cleaved protein was removed through incubation with Ni-NTA beads (Qiagen) for 1 hr at room temp, before the supernatant was concentrated and loaded onto a S75 26/600 gel filtration column. Relevant fractions were pooled and concentrated to between 1 and 20 mg/ml before crystallization trials were set up, and aliquots were flash cooled in liquid nitrogen before storage at -80°C.

Binding of Notch ligands to Ganglioside/Sphingomyelin-rich liposomes

Liposomes were prepared as described in Chillakuri et al., 2013 at a 80:15:5 ratio (w:w:w) of gangliosides/sphingomyelin:PS:PE.

2. Extended structure determination procedures:

General data collection, data processing, and structure determination methods:

Programs included in or affiliated with the *CCP4* software package (Winn et al., 2011) were used for most steps during data processing and structure solution. The collected datasets were indexed, integrated, scaled and merged using *Xia2*. *Phaser* (McCoy et al., 2007) was used to solve the structures by molecular replacement. *Buccaneer* (Cowtan, 2006) was used for automatic model building. The refinement of models was done using iterative steps of model building in *Coot* (Emsley et al., 2010), in *Refmac* (Murshudov et al., 1997), and in *Phenix refine* (Afonine et al., 2012). Final

model validation was undertaken using *Molprobity* (Chen et al., 2010). Images of the refined structures were prepared with the molecular graphics program *CCP4MG* (McNicholas et al., 2011).

Crystallisation and structure determination of human Delta-like4 NE3:

The best diffracting crystal was obtained by vapour diffusion with 3 mg/ml Delta-like4 NE3 protein in 0.1 M MES pH 6.5, 12% (w/v) PEG-20K at a 3:1 protein:precipitant ratio. Delta-like4 was crystallised in the presence of 5 mM $BaCl_2$ as it was expected to bind to calcium. The crystal was cryoprotected with 35% (v/v) glycerol, and data were collected to 2.2 Å at Diamond Light Source, beamline I04-1 (Table 1). The crystal belonged to space group C2 with unit cell dimensions a = 127.9 Å, b = 49.8 Å, c = 70.0 Å, α = 90.0°, β = 109.2°, γ = 90.0°, and with one molecule in the asymmetric unit. This crystal form was solved by molecular replacement using the C2 domain of human Jagged 1 (PDB code = 4CC1) (Chillakuri et al., 2013), before the remaining domains were found using Jagged1 as a search model. The structure was built using the automatic model building software *Buccaneer* (Cowtan, 2006). The structure was refined to final R_{work}/R_{free} values of 0.1823/0.2293 using *Refmac* and *Phenix refine*. All residues in the NE3 construct (27-325) (excluding C-terminal tag) are visible in the electron density. There was no density representing calcium in the electron density maps, suggesting that the C2 domain of Delta-like4 does not bind to calcium. Many of the aspartate residues in Jagged1 that bind calcium are not conserved in Delta-like4 (Figure 2C). Calcium was still not visible in the electron density maps despite soaking with 10mM $CaCl_2$. This is consistent with the structure of rat Delta-like4 in the Notch1-Delta-like4 variant complex structure (Luca et al., 2015), which is not bound to calcium ions. Threonine-299 in EGF3 appears to be O-glycosylated, being at an equivalent position to the site in EGF3 modified in Jagged1 (Chillakuri et al., 2013).

Crystallisation and structure determination of human Jagged2 N-EGF3 and N-EGF2:

The best diffracting crystal was obtained by vapour diffusion with 2.3 mg/ml Jagged2 N-EGF3 protein in 0.1 M Sodium citrate pH 5.3, 20% (w/v) PEG-5000 MME at a 3:1 protein:precipitant ratio. Jagged2 NE3 was crystallised in the presence of 10 mM $BaCl_2$ (in case experimental phases were required). The crystal was cryoprotected with 30% (v/v) ethylene glycol, and data were collected to 2.8 Å at Diamond Light Source, beamline I04-1 (Table 1). The data were anisotropic with the crystal only diffracting to 3.6 Å along one axis. The crystal belonged to space group $P2_12_12_1$ with unit cell dimensions a = 46.8 Å, b = 77.2 Å, c = 96.4 Å, and with one molecule in the asymmetric unit. The structure was solved by molecular replacement using the C2 domain of Jagged1 (PDB ID = 4CC1) using *Phaser* (McCoy et al., 2007), before the remaining domains of Jagged1 N-EGF3 were placed sequentially into the electron density with iterative rounds of rigid body and restrained refinement in *Refmac* (Murshudov et al., 1997). The structure was refined to final R_{work}/R_{free} values of

0.2550/0.3121 using *Phenix refine* (Afonine et al., 2012). Barium was not visible in the electron density maps, and neither were the loops of the C2 domain (residues 52-61, 63-65, and 154-157). The density representing residues 79-85 in the C2 domain is poor, and the final loop of EGF3 is not traceable (residues 335-348). Threonine-322 in EGF3 appears to be O-glycosylated, being at an equivalent position to the site in EGF3 modified in Jagged1 and Delta-like4. The long loop between strands 3 and 4 forms a few helical turns before it extends and interacts with EGF1 of a neighbouring molecule. Due to the absence of density representing the C2 domain loops, crystallization trials were set up in the presence of calcium chloride for both Jagged2 N-EGF2 and N-EGF3.

Jagged2 N-EGF2 was crystallised at 3.4 mg/ml in the presence of 10 mM $CaCl_2$ in 0.05 M potassium dihydrogen phosphate, 20% (w/v) PEG-8000 at a 3:1 protein:precipitant ratio. Crystals were cryoprotected with 20% (v/v) glycerol, and data were collected to 2.7 Å at Diamond Light Source, beamline I02 (Table 1). The data were anisotropic with the crystal only diffracting to 3.5 Å along one axis. The crystal belonged to space group $P2_12_12_1$, with unit cell dimensions a = 48.3 Å, b = 83.9 Å, c = 99.4 Å (similar to the apo N-EGF3 crystal form), and with one molecule in the asymmetric unit. The structure was solved by molecular replacement using the N-EGF2 portion of the apo N-EGF3 model using *Phaser* (McCoy et al., 2007). Although most of the residues in the loops of the C2 domain were visible in the electron density, and there appeared to be electron density corresponding to calcium ions, interpretation of the density only became obvious following structure solution of a different crystal form of Jagged2 N-EGF2 (see below). Despite collecting data at 1.3 Å wavelength to potentially locate calcium ions, no correlation between the anomalous differences was visible. The final structure was refined to R_{work}/R_{free} values of 0.2299/0.2635 in *Phenix refine* (Afonine et al., 2012). Density is visible for all residues apart from some residues in the loops of the C2 domain (52-59 and 107-123). The B factors of the three calcium ions ranges from 67-71 Å^2, which is similar to the coordinating ligands (57-78 Å^2).

Crystallisation trials were set up of the first half of the gel filtration peak (due to different glycosylation forms) of Jagged2 N-EGF2. Jagged2 N-EGF2 also crystallised at 3.3 mg/ml in the presence of 20mM $CaCl_2$ in 0.1 M sodium cacodylate pH 6.5, 0.2 M ammonium sulphate, 30% (w/v) PEG-8000 at a 1:1 protein:precipitant ratio. Crystals were cryoprotected with 20% (v/v) ethylene glycol, and data were collected to 2.8 Å at Diamond Light Source, beamline I04-1 (Table 1). The crystal belonged to space group P1 with unit cell dimensions a = 62.6 Å, b = 92.9 Å, c = 97.0 Å, α = 71.7°, β = 83.2°, γ = 82.7°, and with six molecules in the asymmetric unit. The structure was solved by molecular replacement using the N-EGF1 portion of the above Jagged2 N-EGF2 structure as a search model using *Phaser* (McCoy et al., 2007). The EGF2 domains were then placed into the electron density manually. The final structure was refined to R_{work}/R_{free} values of 0.2299/0.2635 in *Phenix refine* (Afonine et al., 2012). Three calcium ions are clearly bound to the C2 domain in 3 of

the 6 molecules in the asymmetric unit. The B factors of the three calcium ions ranges from 35-61 $Å^2$ (in chain A), which is similar to the coordinating ligands (35-52 $Å^2$). Density is visible for the residues in the loops of the C2 domain apart from residues 57-59 (in chain A). The loop between strands 3 and 4 is longer in Jagged2 than in the other Notch ligands, and adopts a helical conformation. The density for 2 of the 6 molecules in the asymmetric unit (chains A and B) is better than the other four. Asn-153 is N-glycosylated with density representing the first four sugar moieties visible (until the β(1-4)-linked mannose) in at least three of the six chains. The α(1,6)-linked fucose packs against Trp-151 (unique to Jagged2, conserved in rat and mouse Jagged2).

Crystallisation and structure determination of human Notch2 EGF11-13:

Crystallisation trials were set up of Notch2 EGF11-13 in which the C-terminal BirA and His tags were cleaved off using PreScission protease. Notch2 EGF11-13 was crystallised at 20 mg/ml in the presence of 10 mM $CaCl_2$ in 0.1 M sodium cacodylate pH 6.5, 0.2 M sodium acetate, 30% (w/v) PEG-8000 at a 3:1 protein:precipitant ratio. Crystals were cryoprotected with 15% (v/v) ethylene glycol, and data were collected to 1.9 Å at Diamond Light Source, beamline I04 (Table 1). The data were anisotropic with the crystal only diffracting to 2.3 Å along one axis. The crystal belonged to space group $P2_12_12_1$ with unit cell dimensions a = 20.2 Å, b = 49.8 Å, c = 125.5 Å, with one molecule in the asymmetric unit. The structure was solved by molecular replacement using the individual EGF domains of Notch1 (PDB ID = 2VJ3) (Cordle et al., 2008). EGF12 was found first, before EGF11 and EGF13. The final structure was refined to R_{work}/R_{free} values of 0.2278/0.2636 in *Phenix refine* (Afonine et al., 2012). All residues in the EGF11-13 construct (415-532) are visible in the electron density, with each EGF domain bound to one calcium ion. Density representing *O*-glucose on Ser-462 and Ser-500, and *O*-fucose on Thr-470 was clearly visible. Density representing xylose linked to the *O*-glucose on Ser-462 was also visible.

References:

Afonine, P.V., Grosse-Kunstleve, R.W., Echols, N., Headd, J.J., Moriarty, N.W., Mustyakimov, M., Terwilliger, T.C., Urzhumtsev, A., Zwart, P.H., and Adams, P.D. (2012). Towards automated crystallographic structure refinement with phenix.refine. Acta Crystallogr D Biol Crystallogr *68*, 352-367.
Chen, V.B., Arendall, W.B., 3rd, Headd, J.J., Keedy, D.A., Immormino, R.M., Kapral, G.J., Murray, L.W., Richardson, J.S., and Richardson, D.C. (2010). MolProbity: all-atom structure validation for macromolecular crystallography. Acta Crystallogr D Biol Crystallogr *66*, 12-21.
Chillakuri, C.R., Sheppard, D., Ilagan, M.X., Holt, L.R., Abbott, F., Liang, S., Kopan, R., Handford, P.A., and Lea, S.M. (2013). Structural analysis uncovers lipid-binding properties of Notch ligands. Cell Rep *5*, 861-867.
Cordle, J., Johnson, S., Tay, J.Z., Roversi, P., Wilkin, M.B., de Madrid, B.H., Shimizu, H., Jensen, S., Whiteman, P., Jin, B., *et al.* (2008). A conserved face of the Jagged/Serrate DSL domain is involved in Notch trans-activation and cis-inhibition. Nat Struct Mol Biol *15*, 849-857.
Cowtan, K. (2006). The Buccaneer software for automated model building. 1. Tracing protein chains. Acta Crystallogr D Biol Crystallogr *62*, 1002-1011.

Emsley, P., Lohkamp, B., Scott, W.G., and Cowtan, K. (2010). Features and development of Coot. Acta Crystallogr D Biol Crystallogr *66*, 486-501.
Luca, V.C., Jude, K.M., Pierce, N.W., Nachury, M.V., Fischer, S., and Garcia, K.C. (2015). Structural biology. Structural basis for Notch1 engagement of Delta-like 4. Science *347*, 847-853.
McCoy, A.J., Grosse-Kunstleve, R.W., Adams, P.D., Winn, M.D., Storoni, L.C., and Read, R.J. (2007). Phaser crystallographic software. J Appl Crystallogr *40*, 658-674.
McNicholas, S., Potterton, E., Wilson, K.S., and Noble, M.E. (2011). Presenting your structures: the CCP4mg molecular-graphics software. Acta Crystallogr D Biol Crystallogr *67*, 386-394.
Murshudov, G.N., Vagin, A.A., and Dodson, E.J. (1997). Refinement of macromolecular structures by the maximum-likelihood method. Acta Crystallogr D Biol Crystallogr *53*, 240-255.
Winn, M.D., Ballard, C.C., Cowtan, K.D., Dodson, E.J., Emsley, P., Evans, P.R., Keegan, R.M., Krissinel, E.B., Leslie, A.G., McCoy, A.*, et al.* (2011). Overview of the CCP4 suite and current developments. Acta Crystallogr D Biol Crystallogr *67*, 235-242.
