# Supplementary material for: Structural and functional dissection of the interplay between lipid and Notch binding by human Notch ligands
Source: EMBO J. 2017 Jun 1;36(15):2204–15. doi: 10.15252/embj.201796632 (PMC5538765; doi:10.15252/embj.201796632)
Supplement: Supplementary file 2 — Expanded View Figures PDF [file EMBJ-36-2204-s002.pdf]

Expanded View Figures

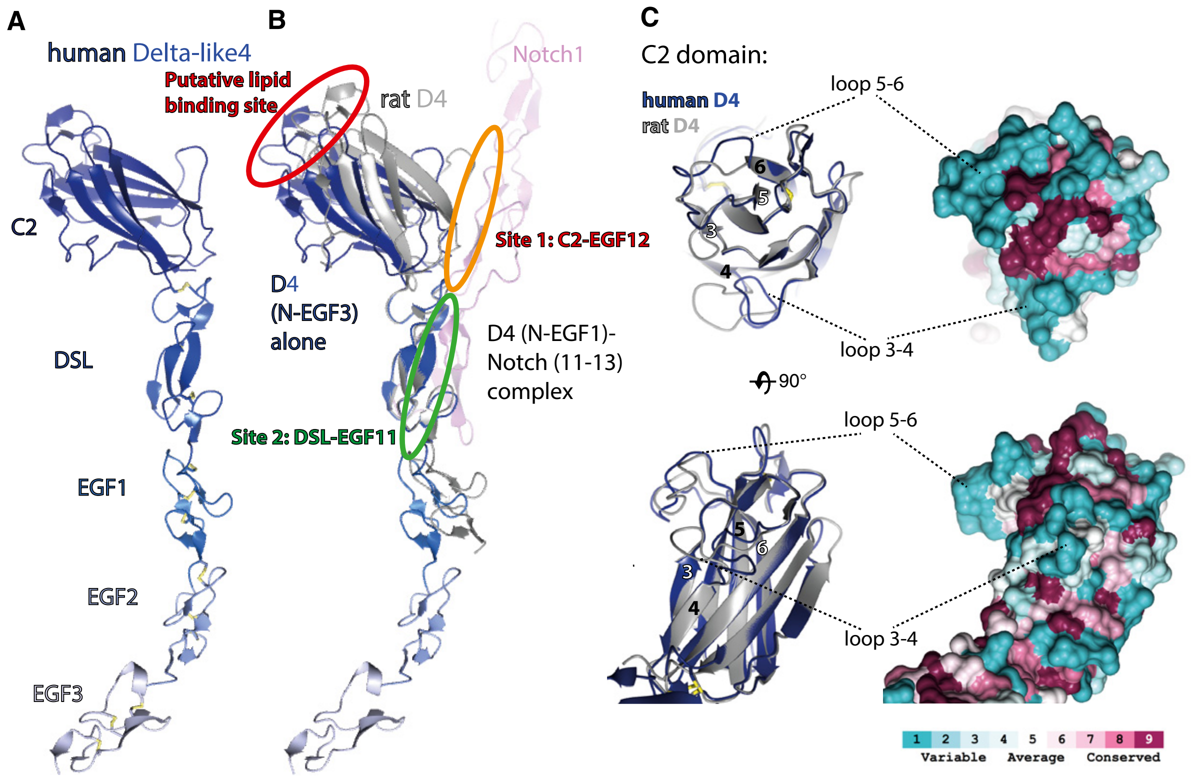

Figure EV1. Structural comparison of human Delta-like4 ligand with rat Delta-like4.

A–C Superposition of human Delta-like4 (N-EGF3) (A) with rat Delta-like4$_{SLP}$(N-EGF1) (grey) –Notch1 (EGF11-13) (pink) complex structure (PDB ID = 4XL1) (Luca *et al*, 2015) across the DSL domain, highlights the greater angle between the C2 and DSL domains in the apo ligand structure (B). There are also differences in the loops between strands 3 and 4 (CBR2), and strands 5 and 6 (CBR3) in the C2 domain between rat and human Delta-like4 (C). The ConSurf Server (consurf.tau.ac.il) was used to create a surface representation of the evolutionary conservation of residues in Delta-like4 based on an alignment from zebrafish to humans using ClustalO (data not shown). This highlights that CBR2 and CBR3 are the most variable regions of the structure (C).

© 2017 The Authors

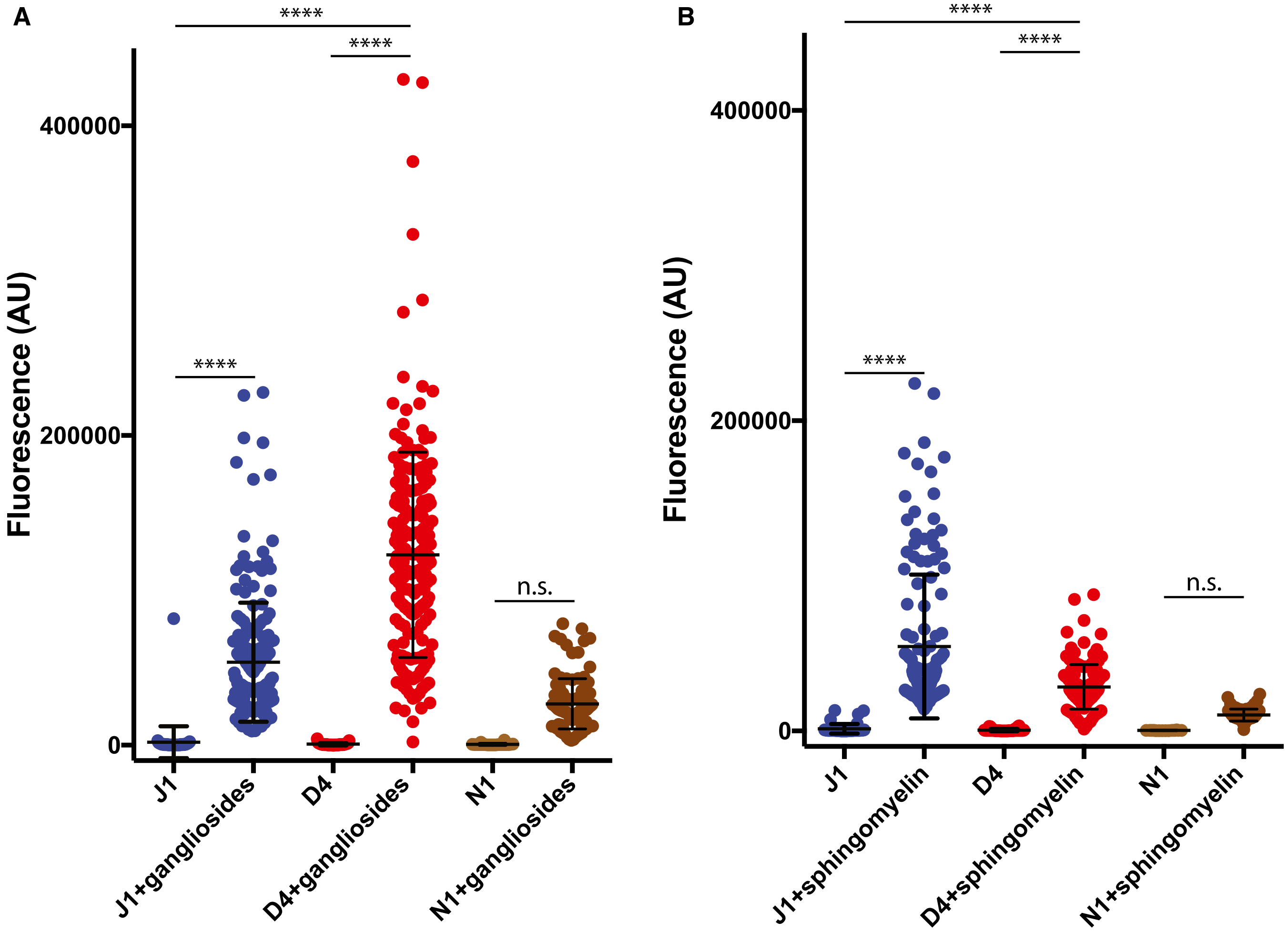

Figure EV2. Human Delta-like4 and Jagged1 exhibit different binding preferences for ganglioside- or sphingomyelin-rich liposomes.

A, B Delta-like4 shows preferential binding to ganglioside-rich liposomes compared to Jagged1 (A) whilst the converse is true for sphingomyelin-rich liposomes (B). Seventeen and nine independent experiments were performed (respectively) with a minimum of twenty replicates in each. Data were analysed with Prism 6 or 7 (GraphPad, San Diego, CA, USA). Comparisons between two groups were performed with a two-tailed unpaired *t*-test. Statistical differences among various groups were assessed with ordinary one-way ANOVA by comparison to the mean of a control column. Values are presented together with the mean ± SD. ****$P < 0.0001$.

 © 2017 The Authors
